# Resiliency with Forced Migrants: A Qualitative Study of Providers and Forced Migrants through a Resilience Perspective

**DOI:** 10.3390/bs12020027

**Published:** 2022-01-26

**Authors:** Nicole Dubus

**Affiliations:** School of Social Work, College of Health and Human Science, San Jose State University, San Jose, CA 95192, USA; nicole.dubus@sjsu.edu

**Keywords:** DACA, ecological theory, resettlement, resilience, forced migration, social work

## Abstract

In the last ten years, the world has experienced unprecedented, forced migration due to civil unrest, political persecution, and the ever-growing climate crisis. This is a qualitative study of the professional experiences of social workers (*n* = 73) working with forced migrants (*n* = 34) and the lived experiences of forced migrants in several countries: Germany, Greece, Iceland, Mexico, Switzerland, and the United States. Social workers reported that most of their interventions involved short-term case management that focused on securing initial housing and healthcare. Cognitive Behavioral Therapy (CBT) was the primary intervention for behavioral health issues. The recipients of these services were appreciative of the pragmatic approach of case management as it helped them meet concrete needs. When resiliency enhancing interventions were used, recipients reported a greater sense of self-control, greater optimism for the future, and less anxious symptoms. The resiliency model used is discussed. This is a possible universal approach to working with forced migrants.

## 1. Introduction

Approximately 1 in 95 people in 2021 had to flee their homelands due to various unsafe situations. Currently, over 82 million people worldwide are forced migrants [1]. Of this number, more than 26 million are refugees. A refugee is a term for people who have been forced from their homeland due to persecution and civil unrest. After WWII, the United Nations developed universal human rights and a policy to assist in resettling displaced individuals and families. The founders of the 1951 convention that established the refugee program expected the program to last no more than three years. More than 60 years later, this program continues to help refugees worldwide.

The United Nations High Commissioner for Refugees (UNHCR) designates who is considered a refugee based on the situations in their country. Once designated, refugees are eligible to receive assistance from UNHCR, such as services within refugee camps. If it remains unsafe for the refugee to return home, UNHCR facilitates resettlement into another country.

Fleeing a country for safety is a highly stressful event. If one is designated as a refugee, they may receive support and assistance in the host country. However, those who had to flee their homelands but do not qualify as a refugee according to the UNHCR are labeled asylum seekers when they arrive at the border of the new country. Asylum seekers often receive very little assistance, if any, while they wait to hear if they can stay. This process can take months or years [2,3,4,5]. There are other forms of forced migrants. Some people who travel across borders are undocumented and try to stay and find jobs for employment without being discovered and deported. Then, there are climate refugees. This term describes those who have had to flee their homelands due to severe climate changes that have made their homelands uninhabitable. Unfortunately, this is a fast-growing group. For this article, “forced migrants” include refugees, asylum seekers, undocumented migrants, and climate refugees. This study examines the experiences of those who have been forced to flee their homeland and those who work with forced migrants to assess culturally effective health and behavioral health services. The needs of forced migrants can be varied and complex. This study posits that enhancing resilience might be a universally effective intervention for a population that has undergone severe stress and trauma.

### Health and Behavioral Health Needs

Health and behavioral health are terms used to describe physical health and mental health issues. Forced migrants differ from immigrants in several ways. Each term describes a person who leaves their country for a new country. However, the term immigrant implies a certain amount of choice for the person moving, whereas the term forced migrant suggests that the person would not move if they did not have to. This small distinction represents vast differences between the group’s resources and experiences [1,2,6,7].

The following are useful examples in fleshing out the fundamental differences. A medical doctor, for example, may decide to live and work in the United States for professional or personal reasons. This person enters the United States with a career that they can transfer to the new country, most likely with some connections to colleagues or family who also live in the United States. In their homeland, they had the means to obtain healthcare and to study English or other languages.

A forced migrant, on the other hand, feels desperate and hopeful to find safety or work. The conditions in their homeland make it dangerous or unlivable, and they move through borders in their search for safety. They may arrive at the border without a promise of employment, no money to secure housing and very few resources to aid them. Alternatively, they may be invited into the country through United Nations refugee programs, given temporary housing, some language classes, and information on job searches. Most jobs offered are for unskilled physical labor [8,9].

For many refugees, their struggles did not begin at the border. Instead, they may have come from a refugee camp, where they stayed for months or years. Life in these camps may have been difficult, with unsanitary conditions and continued fear of violence. Before reaching the camps, they may have been abused, tortured, and/or confined [10,11,12]. They have all experienced loss: loss of family members, loss of their homes, and loss of their country. When a forced migrant arrives at the border, they likely have health and behavioral health needs in addition to their financial needs. In contrast, when an immigrant arrives at the border, they are more likely to seamlessly integrate into their new country.

Community health centers and NGOs are on the frontlines of providing services to forced migrants. Nationally and internationally, this is a challenge. Services are often provided in silos, separate from other services and agencies. This can cause gaps in the service provision and redundancies that are inefficient for the limited resources of the agencies [13,14,15,16,17,18,19,20]. Agencies and communities may have very little notice of the forced migrants’ arrival and therefore scramble to find interpreters and translators. Many will arrive without medical records; much of their past and reasons for fleeing will be unknown. These factors create challenges for providers as the staff tries to provide culturally effective services for an unknown population [21,22,23,24].

Studies have examined the health and behavioral health services provided to forced migrants [25,26,27,28,29,30,31,32,33], p. 69. Housing, learning the host country’s dominant language, and obtaining a job are prevalent needs reported by forced migrants and the agencies working with forced migrants [28,29,30,31,32,33]. Studies found that most health settings provided initial physical assessments [34]. Some settings offered ongoing healthcare and had social workers and counselors available. Research on best practices for working with forced migrants describe several challenges, including difficulty finding interpreters and translators for different languages, lack of health records for the patients, cultural differences in what causes illness and what cures it, post-traumatic shock in patients who experienced abuse and neglect, lack of transferable education for the newcomer to work at the professional level they had in their home country, and a short time in which to provide help because most assistance from programs end after the first year of resettlement. Behavioral health providers primarily used cognitive and behavior therapy, case management, and referral to other programs [35,36,37]. Post-traumatic stress disorder, depression, and anxiety are highly prevalent. Many forced migrants present with illnesses and injuries that pre-dated their exodus [10,35,36,37,38,39]. Health and behavior health providers feel ill-prepared for each group’s arrival [40,41,42]. They reported difficulties of not knowing if someone had ever been immunized or if they had allergies to medications. Over time, they learned about each group’s background and the conditions that forced them to flee. However, the providers wanted to know this before they arrived. Behavioral health providers described frustrations because they did not have an interpreter available, did not know their culture or history, and felt too overwhelmed to take the time it would take to individualize treatment.

Behavioral health, for many cultures around the world, is unfamiliar [41]. To be effective, behavioral health providers need to create trust. They do this by engaging, listening, showing concern, and being clear about what they can provide and through repetition of these actions over time. For clients whose culture is unknown to the provider, the provider must take more time to learn from the client. With clients who have PTSD, the provider must take extra care and proceed with a trauma-informed approach [10,35,36,37,38,39,43,44,45,46,47,48,49,50].

BHPs reported primarily using CBT. Most providers also stated that they did not know other approaches well enough for use with trauma survivors. CBT is considered an effective treatment approach for depression and anxiety [35,51,52]. However, depression and anxiety are shaped and understood through cultural lenses [53,54,55,56]. How symptoms are expressed, and the meaning of these symptoms can vary among cultures. To be effective, a provider needs to know what these symptoms mean for their client/patient [57,58,59,60,61].

Resilience is an internal experience that can be enhanced by helping the individual identify and access resources. Resilience has been examined as a set of personal traits, skills, and coping techniques seen in one’s ability to obtain resources and support [62,63,64,65,66,67,68,69,70,71,72,73,74,75,76,77]. It can be assessed and enhanced. In assessing for resilience, one needs information on the individual’s baseline functioning before the disruptive event. As the individual describes the disruptive events, the provider listens for patterns, examples of skills, and themes in the narratives. When the individual recounts details of their recent stressful events, the provider reminds the individual of past stresses that they overcame and gives them an example that they had previously shared.

Resilience can be enhanced through social support, which could include family and friends. Resilience can be recognized in others by the person’s ability to identify their needs; help others; temper stress; and initiate tasks that move the person forward, passed the stressful or traumatic event(s) [78,79,80,81,82,83,84,85,86,87,88,89]. Resilience can be measured by metrics that reflect the person’s ability to function as well as they did before the stressful event.

Resilience can be enhanced. When a neighborhood is bombed and there is no electricity or plumbing for days, residents can be isolated from everything outside of their block. A behavior health provider or community member could organize supply swaps where residents gather their surplus goods and trade them with those of their neighbors; a couple of people could help the neighborhood assess each household’s needs, and vulnerable residents can be identified and supported. These interventions enhance each person’s resilience while also enhancing the community.

Resilience can be the focus of interventions. As described earlier, cognitive and behavioral interventions focus on the unwanted thoughts and behaviors of an individual. Resilience interventions focus on identifying and enhancing the individual’s resilience while also identifying and fighting against oppressive institutional barriers.

In working with forced migrants, a resilience perspective perceives the individual in a geopolitical context, and, within this context, the countries involved, the cultures, international and domestic systems of oppression and privilege, and the resources available. There are important changes in how resilience is understood, measured, and addressed [60,61,62,63,64,65,66,67]. Borrowed from physical science and, later, cell biology, resilience in behavior science has shifted from resilience being a personal characteristic to a reflection of a person’s lifelong risk and protective factors [78,79,80,81,82]. Poverty is a risk factor. Public assistance is a protective factor. Having citizenship is a protective factor. However, the existence of protective factors and risk factors does not tell the whole story of resilience. The whole story of resilience is in how people use the resources available, how they respond to crises and change, and what coping skills they use to normalize their lives when their world is turned upside down.

This project sought culturally effective interventions for use with forced migrants. Enhancing clients’ resilience may be an effective intervention that is relevant and universal. To explore this further, this project analyzed 107 interviews with behavioral health professionals (*n* = 73) and adult forced migrants (*n* = 34) from five countries (Germany, Greece, Iceland, Mexico, Switzerland, and the US).

## 2. Methods

### 2.1. Original Study

The data used for this study were collected from a previous qualitative study that examined the professional experiences of providers of services for refugees and forced migrants’ experiences of receiving these services. This study sought to identify culturally effective health and behavioral health services. The selection criteria included being current health or behavioral health provider of persons who can be considered a forced migrant or being a self-identified forced migrant of adult age. Recruitment used key informants to recruit the initial sample of participants. After the initial forced migrant recruitment, subsequent recruitment used snowballing. At the end of recruitment, there were 73 provider participants and 34 forced migrants. All the providers were formally trained as social workers or equivalent profession. The forced migrant participants were refugees (*n* = 21), asylum seekers (*n* = 7), and undocumented migrants (*n* = 6). All forced migrant participants were adults, with the youngest being 22 years old and the oldest being 88 years old; the median age was 53.

#### 2.1.1. Original Study Methods

This study used transcribed interviews with providers (*n* = 73) who work with forced migrants, and with the forced migrants (*n* = 4) who received those services. These interviews were collected by the principal investigator (PI) and were part of a larger study that examined the professional experiences of providers who work with forced migrants and some of the individuals who received services as forced migrants. The forced migrant participants and the providers did not necessarily know each other.

#### 2.1.2. Original Data Collection

The interviews were collected over five years, from 2014 to 2019, in Germany, Greece, Iceland, Mexico, Switzerland, and the United States. The participants had been recruited from contacts of the PI who provided services to forced migrants. Snowballing was used to recruit more participants. Interviews were conducted after informed consent was obtained and conducted in private locations of the participant’s choosing, most often at an office or in the home of the participant. The interviews were audio-recorded for later transcription with the participant’s consent. All interviews of providers (*n* = 73) were conducted in English. Of the forced migrant participants, 22 were conducted with an in-person interpreter and 12 were conducted in English without an interpreter. The data collected were analyzed using thematic content analysis [82]. The PI and two research assistants read the transcriptions first before coding. The transcriptions were re-read, and coding began with in vivo words and passages. This was performed to capture the participants’ own words. In subsequent readings, codes were developed and compared with the other coders and consolidated or refined based on a discussion among the coders. Through this inter-rater reliability check, codes were established. Some codes were combined when the combined code more accurately reflected the participant’s meaning. The codes were compared among all of the interviews and consolidated as appropriate, and larger categories that captured the major themes that emerged were created. The final themes were shared with a key informant from each country to ensure the findings were accurate.

### 2.2. Methods of the Current Study

This study used anonymous data from the original study. The PI and two research assistants who were not involved in the larger research examined the transcripts. Similar data analysis was conducted, whereby the transcripts were read and then read again, with each reader coding for resiliency this time. A resilience coding system was developed before the analysis began. The PI and research assistants based the coding system on resilience literature. The coders were instructed to highlight words and phrases in the transcripts that address concepts of resilience. Once the transcripts were coded, the codes were reviewed and discussed, combined, or changed to make the codes more accurate. Once the codes were agreed upon, they were reviewed for possible larger categories. The final categories were (1) used own resilience to help the forced migrants (examples of when a provider referenced their past challenges in trying to understand and help clients), (2) enhancing resilience in clients (when a provider deliberately chose interventions to enhance resilience in clients separate from the work of referring clients to resources or services and providing cognitive behavioral therapy), (3) non-resilience interventions, and (4) demonstrations of resilience by the forced migrant (examples of skills and behaviors of the participants that demonstrated resilience). Once categories were created, the categories were reviewed to assess for overarching themes from the findings. The themes are discussed below (Table 1).

## 3. Results

Social workers reported that most of their interventions involved short-term case management that focused on securing initial housing and healthcare. Cognitive Behavioral Therapy (CBT) was the primary intervention for behavioral health issues. The recipients of these services were appreciative of the pragmatic approach of case management as it helped them meet concrete needs. However, recipients who suffered from Post-Traumatic Stress Disorder or who did not plan to resettle in that host country expressed frustration. They reported that CBT was not helpful. When resilience-enhancing interventions were used, recipients reported a greater sense of self-control, greater optimism for the future, and less anxious symptoms.

There were two major themes of resilience that captured the experiences of the providers and forced migrants: (1) the provider’s resilience; (2) forced migrant’s resilience.

### 3.1. Providers’ Resiliency Helped Them Help Others

The providers all had social work training or its equivalent. Each provider practiced cognitive behavioral therapy. In working with forced migrants, providers had new challenges.

#### 3.1.1. Challenges for Providers

Providers (69/73) stated that it felt like they were starting all over again each time a new group came. They described layers of challenges. There were challenges in providing written and verbal language interpretations for all forms and educational materials. Most clients did not come with medical records, and some countries do not record every birth, leaving large gaps in the biopsychosocial history of the clients. There were challenges in bridging cultural differences. This challenge manifested in several ways. Providers (73/73) reported difficulty in addressing mental health issues when the client’s culture does not talk openly about one’s feelings and needs. Providers (48/73) worked at busy clinics and were frustrated when families arrived hours late for an appointment. One provider stated,

Two or more families would come in together like they drove together or something. They would come with food and make a spread in the waiting room. We would have to tell them to pack up and leave, that their appointment was hours ago (provider, female, US).

While there were challenges for every provider in the study, some of the providers (23/73) appeared to have flexible expectations of clients based on cultural differences. One provider shared,

I’ve traveled to foreign countries before so I think that helps me. I ask a lot of questions about their culture, even if I am familiar with it. I mean, I want to know what their culture is to them. And that can be different for each person within the same culture (provider, female, Germany).

The providers’ responses suggest that personal experiences with different cultures enhance a provider’s ability to individualize service delivery. For example, a provider in the United States stated,

We are thrown off our normal schedule when we have new arrivals who have never been to another country. We did this cool thing that I wish we would do more of. We couldn’t get refugees from one of the countries, Buthan maybe, to get here on time. Made the staff so mad. So then we decided it would be easier to change our ways than to have them change theirs. So we started a drop-in clinic program. Every Wednesday afternoon we reserved for walk-ins. I think many just thought that’s how we do it. That we are only open on Wednesdays. However, for us, one day a week was a drop in and every other day was normal scheduling. It worked really well (provider, female, US).

#### 3.1.2. Provider Using Own Resilience in Enhancing Resilience in Others

Throughout the interviews with the providers, a theme of “provider’s resilience” emerged. All of the providers practiced CBT with their clients. Of the 73 providers interviewed, 19 providers (1 German, 7 Greek, 2 Icelandic, 4 Mexican, 1 Swiss, and 4 American) demonstrated resilience in working with clients. The same concepts used to code for resilience were used to identify providers who appeared to respond to the client with greater empathy, used strength perspective in assessment and treatment, and spoke about their own experiences with profound stress and dislocation. Those who described their challenges that were relatable to their clients’ experiences shared what helped them during those times. These providers drew most clearly from their own lives, as this female provider from Greece describes, “I’ve never been a refugee. No. However, I have gone through times where my whole life was torn apart. I can relate to that”.

Providers who related to the clients also were more likely to suggest interventions that the other providers did not. For example, a provider from the US stated,

With this one family, learning English now was just too stressful. So, I didn’t push that. Instead, we spent a good amount of time talking about their journey and what helped them survive. Could they use those same skills here? I think feeling safe at this point is more important than knowing English. I’m a bit alone in this opinion (provider, female, US).

Other providers who seemed to use their resilience in working with clients tended to develop treatment plans focused on the family’s strengths. Working with clients this way took more time. For example, this provider describes how his work is different from his coworker who also works with forced migrants. These two providers worked with refugees from various countries in Africa.

“She gets a new client and after that first session they are hooked up to everything”. He laughs. “Me? I’m still talking to them about their life back home…it takes that time to get to know them. What happened back home? What skills do they have? Can they use those skills here?” (Male, Provider, Germany).

Providers who reflected on their own experiences with moving through hard times seemed to bring the same to the narratives of the clients. How did they manage past stress? How do the clients perceive the recent changes in their lives?

### 3.2. Forced Migrants’ Resilience

Analyzing the forced migrants’ transcriptions through a lens of resilience revealed insights into their strengths that were not revealed when analyzed for a previous study that examined their needs. For example, one participant described going to the social worker’s office most mornings. As he described, “I wanted her to remember me and my family. If there was a benefit we could get, I wanted them to think of us” (male, refugee, Greece). This behavior had been described by a worker in a previous study as demanding and stressful for the worker. In this study, the same behavior was coded for resilience as it demonstrated his ability to assess his own needs and to proactively seek out support. In the previous study, a forced migrant refusing language classes was perceived as non-compliant or “entitled”. In this study, when a participant declined language classes, the transcripts were reviewed further in context to see if there were explanations as to why the family was not engaged in language classes. This deeper analysis yielded stories of families consumed with PTSD and unable to attend language classes, families that never wanted to leave their homelands and are hoping the migration is temporary, and individuals with traumatic brain injuries or anxiety who are unable to engage in the classes.

The transcripts contained passages that were never coded from the previous study. In this study, these passages helped understand the forced migrants’ resilience. Forced migrant participants reported talking to their family and friends via social media apps on their phones and computers. This was mentioned by the social workers as well. One female social worker from Iceland commented, “I go to their homes. They are online with their families. I have never been to families’ homes and not seen them chatting online.” This appeared concerning for some of the providers as seen in this quote,

They don’t come to language classes. Why should they? They stay home and talk in their native languages online. They watch shows from their country. I think we will get them to learn our language if they can’t speak theirs all day (female, provider, US).

When these passages were coded for resilience, establishing reliable connections with distant family members was perceived as demonstrating resilience. Forced migrant participants described “holding on” to their culture through teaching their native language, food, music, and traditions to their children and by keeping in touch with family who may be scattered around the world. An important element in resilience is the ability to reach out for support and to maintain supportive relationships with friends and family.

#### Multigenerational Resilience

Many of the forced migrant participants were members of a large family. Grandparents resettled with members of their families. The forced migrant grandmothers seemed to have a different experience with resettlement than the rest of their families. They were more aware of civil unrest in their homelands years before their children. They had lived longer and had many experiences of overcoming adversities. From this, they developed resilience, which served them well during the resettlement. They knew terror and how to move past it; they expressed more confidence in surviving stressful times; they knew more what they needed to feel safe, and they were not as invested in future goals. They were more concerned for their family and less about their futures. They remained in the apartment, cleaned, cooked, and cared for the children after school. When they left the apartment, it was with a family member. For these participants, learning the country’s language was not essential. In addition, many felt overwhelmed at learning a new language.

The grandfathers fared less well. While the younger generations in the family were focused on finding work and learning the language, the older men seemed sad. They did not work and were not expected to due to their age, yet they wanted to feel helpful and competent. There was little for them to occupy themselves with at home, and they did not need to work outside of the house. Resilience is being able to return to one’s previous level of functioning before disruptive events. The grandfathers had a certain amount of respect in their home country and within their families. That social capital seemed to vanish when they resettled.

A person’s ability to adapt and find resources to help them function in their new environs is a skill of resilience. Some of the grandfathers were able to find a temple, mosque, or church. This lessened their isolation and sadness. “I go to the mosque every Friday night. Just men like me. We talk and share stories (male, 70, forced migrant, Iceland)”.

Forced migrant participants described their young sons joining soccer teams at school. This activity was familiar to the boys and allowed them to play with other children even if they could not communicate in the same language. This was notably different for the girls. Families stated that their daughters came directly home from school and helped cook and clean. This was most true for forced migrants who came from cultures where females were less likely to play sports and more expected to become caregivers to their families. Parents shared that some of the girls had a more difficult time adjusting to their new lives. They reported feeling isolated and lonely. One grandmother discussed her granddaughter.

We share a room. I see her try to hide her crying. She doesn’t want to sleep with her grandmother. She wants to make friends and have boyfriends. However, no one knows our language. Girls her age here don’t know us, our culture. I am glad she helps her family, but I am sad for her.

Sports allowed the boys to engage in an activity they enjoyed and felt competent in, and provided opportunities to make new friends and therefore to have social support in their new country. The girls had fewer opportunities to socialize with children their age. They were able to continue with caregiving tasks that they had performed with their families before resettlement. However, they were not provided opportunities to meet their social needs as readily. This appeared to impact their resilience as parents noticed the girls’ sadness.

## 4. Discussion

### 4.1. The Need

It is imperative that those who provide services to forced migrants and policymakers who seek to develop humane policies that help forced migrants find culturally effective treatment approaches. It is challenging for health centers to address the needs of groups of newcomers. Centers need time to prepare to be culturally effective, and providers need to establish reliable interpretive services, to become familiar with global events that push people from their homelands, to become aware of health and mental health issues that might be relevant to the migrant’s situation, and to provide effective evidence-based service delivery.

#### 4.1.1. Provider’s Practice Skills

Many providers used cognitive-behavioral treatment to address the behavioral health issues of the forced migrants. Most providers used case management skills to connect migrants with services. Some providers spent more time listening to the migrant’s story of life before fleeing, during the journey, and their hopes now that they are someplace safer.

These providers expressed an interest in the family’s strengths, how they managed past stress, and how best to move them in the direction of their stated hopes. This meant that, for some migrants, the provider was helping them relocate to a different country, return to their country, or find their own pace during resettlement.

This approach is intriguing. The focus of treatment is less about helping families obtain secured housing, begin working, enroll children in school, or master a new language and more about enhancing the migrant’s resilience. The difference may appear subtle, but it seems that this approach helps forced migrants reflect on their experiences, assess their strengths, and obtain a locus of control over the next steps in their lives.

#### 4.1.2. Enhancing Resilience

Resilience-enhancing interventions can help providers be mindful of the migrant’s experiences and perspectives, an essential element in cultural competency. From this stance, the provider can individualize treatment to the needs of the migrant while focusing on strengths and helping shift the locus of control back to the migrant.

In training providers to work with forced migrants, resilience-enhancing techniques can be taught to providers from all allied health fields, require less training than cognitive behavior techniques, and may address cultural differences in ways CBT cannot. For example, it is common for forced migrants to experience depression or anxiety. CBT is quite effective in treating these conditions. However, not all cultures share the same understanding of what constitutes depression or anxiety. Additionally, CBT focuses on the provider assessing the thoughts and behaviors of the client that are perpetuating their negative state and on coaching clients to think differently and behave differently. This is further complicated with the use of interpreters. In CBT, a technique often implemented is to use the person’s own words to emphasize how certain thought patterns affect one’s mood. When working with interpreters, it may not be possible to use a client’s exact words and meaning; cultural differences can become lost as the interpreter tries to capture the meaning for the provider and the provider perceives the interpreted meaning through their cultural lens. On the other hand, resilience enhancing techniques are based on a deeper understanding of the client’s strengths and experiences.

#### 4.1.3. Using the Providers’ Own Resilience

Interestingly, the resilience of providers might also be helpful. It is possible that provider training could include self-reflection about one’s resilience and strengths, which may help providers assess and enhance others’ resilience. Greene [68,77], described the resilience-enhancing stress model (RESM). It is a metatheory that draws from the life course perspective, ecological theory, and narrative therapy. It could prove culturally effective and teachable to diverse providers working with an ever-changing demographic.

### 4.2. Limitations of Study

There are limitations within this study. Much more research needs to be conducted before it is known if enhancing resilience is an effective approach to working with forced migrants. Research would also need to assess if case management and enhancing resilience are a more universal, culturally effective approach to helping forced migrants than case management and CBT. While this study looked at different countries, the professional experiences of providers, and the personal experiences of forced migrants, it is unable to determine which interventions are most helpful. The data used were from a larger study that, in general terms, examined the needs and experiences of providers and forced migrants. Even though new findings were revealed using these data, a study that specifically tests CBT against RESM is needed. The investigator of this study is familiar with RESM. Other researchers might find different interventions that do not focus on enhancing resilience to be more effective for this population. A limitation of this study is that only certain countries were included. This can limit the scope and universality of the findings. However, the diversity of all of the participants reflects a form of universality that is a strength of this study.

## 5. Conclusions

Providing effective health and behavioral health services to forced migrants is challenging for many reasons. Providers receive groups of newcomers without training about their culture, history, or experiences. This study suggests that focusing treatment on resilience might be helpful. Further research should explore best practices for enhancing resilience. When resilience is strong, people can better make decisions about their future.

## Figures and Tables

**Table 1:** Demographics of participants.

	Median Age	Country of Origin	Male	Female	Status
Provider	NA	Germany, Greece, Iceland, Mexico, Switzerland, and the United States	22	51	NA
Migrant	53	Cambodia, Eritrea, Honduras, Iraq, Sudan, Syria, and Vietnam	20	14	Asylum seekers (7), refugees (21), and undocumented (6)

## Data Availability

Due to the vulnerable populations discussed in these studies, the qualitative data obtained will not be made public.
